# Supplementary material for: Predicting visual function by interpreting a neuronal wiring diagram
Source: Nature. 2024 Oct 2;634(8032):113–23. doi: 10.1038/s41586-024-07953-5 (PMC11446822; doi:10.1038/s41586-024-07953-5)
Supplement: Supplementary file 6 — CRF and ERF predictions for individual TmY4 and TmY9 cells. Analogous to Supplementary Data 3, but for TmY target types. Shown are the top four monosynaptic pathways, the strongest pathway passing through each of the top ten intermediary types (ranking from Extended Data Fig. 7), and the trisynaptic pathway Tm1–TmY–Dm3–TmY (see the section entitled Prediction of spatial normalization). [file 41586_2024_7953_MOESM6_ESM.zip › DataS4/TmY9q/720575940638344511.pdf]

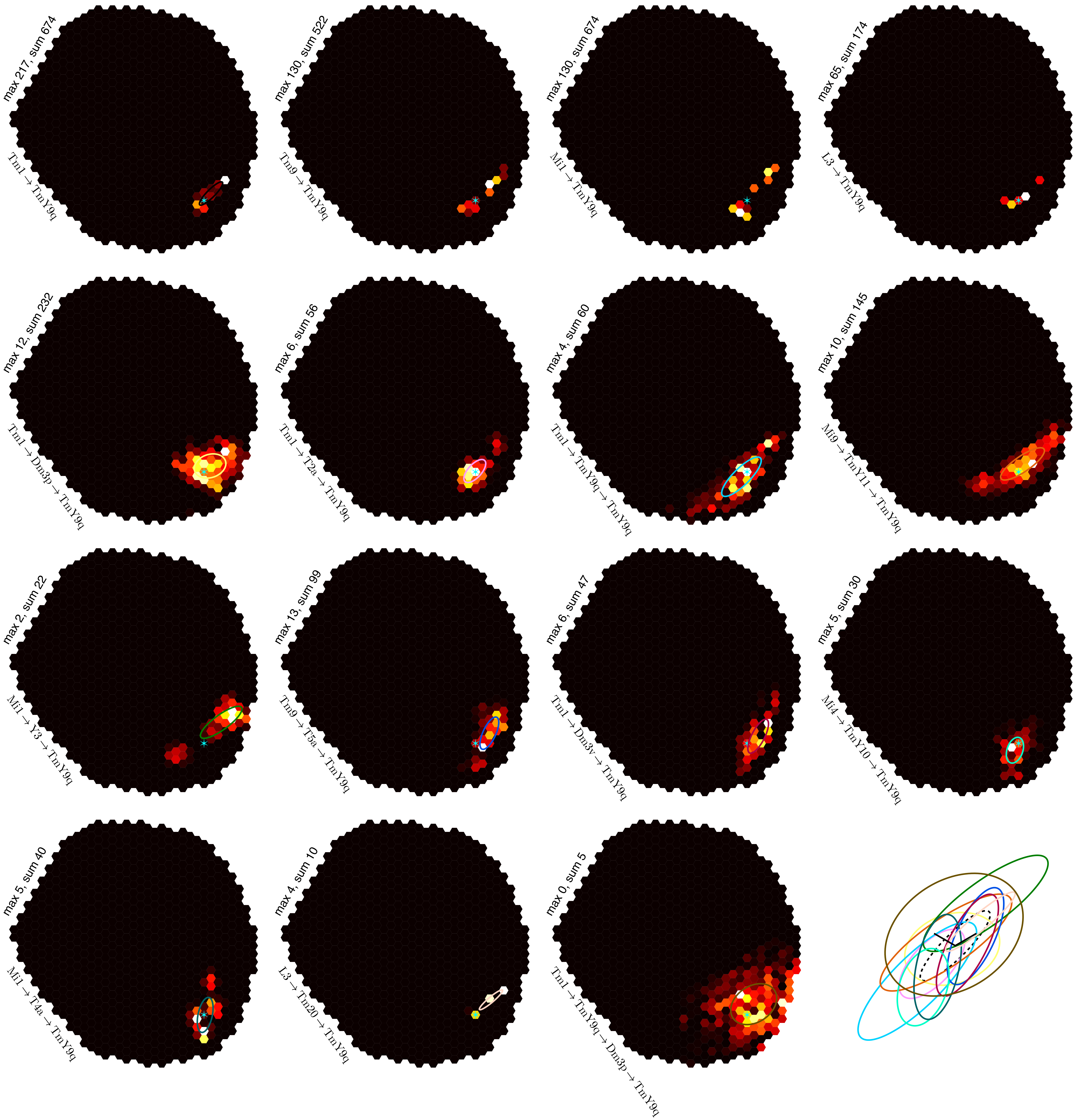
max 217, sum 674
Tm1 → TmY9q
max 130, sum 522
Tm9 → TmY9q
max 130, sum 674
Mi1 → TmY9q
max 65, sum 174
L3 → TmY9q
max 12, sum 232
Tm1 → Dm3p → TmY9q
max 6, sum 56
Tm1 → T2a → TmY9q
max 4, sum 60
Tm1 → TmY9q → TmY9q
max 10, sum 145
Mi9 → TmY11 → TmY9q
max 2, sum 22
Mi1 → Y3 → TmY9q
max 13, sum 99
Tm9 → T5a → TmY9q
max 6, sum 47
Tm1 → Dm3v → TmY9q
max 5, sum 30
Mi4 → TmY10 → TmY9q
max 5, sum 40
Mi1 → T4a → TmY9q
max 4, sum 10
L3 → Tm20 → TmY9q
max 0, sum 5
Tm1 → TmY9q → Dm3p → TmY9q
